# Participant Experience of Taking Part in Periodontal Experimental Studies

**DOI:** 10.1155/ijod/8888815

**Published:** 2024-11-27

**Authors:** Yi-Chu Wu, Fernanda L. Schumacher, Dimitris N. Tatakis

**Affiliations:** ^1^Division of Periodontology, College of Dentistry, The Ohio State University, Columbus, Ohio, USA; ^2^Division of Biostatistics, College of Public Health, The Ohio State University, Columbus, Ohio, USA; ^3^Department of Periodontics, School of Dental Medicine, Case Western Reserve University, Cleveland, Ohio, USA

**Keywords:** mouth mucosa, patient participation, patient satisfaction, surveys and questionnaires, wound healing

## Abstract

**Objective:** Despite the plethora of published periodontal interventional and experimental studies, there are no reports evaluating the experience of the participants as a research subject or their willingness for repeat participation in a similar study. This study aimed to determine the participants' experience and willingness to participate again in periodontal experimental studies and to explore factors that affect the participants' research experience.

**Materials and Methods:** Questionnaires from four completed experimental wound healing studies with 76 total participants were extracted and analyzed. The participants answered the same specific questions at their last study visit. The questions asked were “overall experience in the study” and “willingness to participate in the study again,” with each question providing five levels/possible answers. Questionnaires also provided an opportunity for participants to offer open-ended comments about their participation. Frequency distribution and logistic regression analyses were performed to evaluate the association between the participant's responses and study characteristics.

**Results:** All 76 participants answered the specific questions. Overall, 88.2% of participants had a positive experience from their research participation, and 65.8% of them would participate again in such a study. Of the 76 participants, 50 were in studies that included multiple (≥2) experimental wounds, while 26 received only a single experimental wound. Participation experience was negatively associated with the number of wounds received (*p* < 0.001), while willingness to participate again was positively associated with participation experience (*p* < 0.001).

**Conclusions:** Within the present study limitations, volunteers participating in periodontal experimental studies have an overall positive experience and express willingness for repeat participation.

## 1. Introduction

Improvements in healthcare outcomes have been attributed to several factors, including technological advances [1, 2] and the application of evidence-based practice principles [3–6]. Evidence-based dentistry is an approach that judiciously integrates systematically assessed clinically relevant scientific evidence with the provider's expertise and the patient's needs and desires [7]; this approach has been adopted by national dental organizations [8] and is considered the gold standard to follow.

There is a hierarchical system to assess the strength and quality of research evidence [9, 10], with systematic reviews and meta-analyses considered the highest level of evidence. However, these critical appraisals of the literature require quality studies, which are possible only when the challenges of patient recruitment and retention have been overcome [11, 12]. These challenges are particularly crucial for experimental clinical studies, where volunteer participants do not receive a direct benefit from their participation. Therefore, it is important to document the experience of persons who participate in experimental studies and to identify factors that impact their decision to enroll and participate in such a research endeavor.

There is little information in the literature on participants' experience of taking part in a clinical trial [13, 14] and even less when it comes to participation in dental research studies [15, 16]. The two published scoping reviews on participants' experience both concluded there is scant literature on the topic and that this aspect of clinical trials is seriously under-researched [13, 14]. Except for the reported general finding that potential dental study participants are more reluctant to take part in studies involving any type of surgical/invasive procedure [15], no other information pertaining to periodontal research studies was identified; the only other published study on the attitudes of dental trial participants analyzed the responses of patients who participated in a dental product clinical trial [16]. Therefore, the aim of the present study is to evaluate participants' experience in experimental periodontal clinical studies, their willingness to participate again, and to explore the factors that affect the participants' research experience.

## 2. Methodology

### 2.1. Study Design and Study Population

Questionnaire data from four experimental clinical studies approved by The Ohio State University Institutional Review Board (protocols 2009H0283, 2012H0052, 2018H0323, and 2021H0300) were collected and analyzed. The four studies [17–20] were chosen because of similar nature and participant encounters; they recruited systemically and periodontally healthy adult volunteers and included the creation of wounds in the palate or the buccal gingiva, solely for research purposes. The participants of the four studies received no direct benefits from participation, a fact stated in each study consent form. Only deidentified data were available for data extraction and analysis. For all four studies, the questions pertaining to participation experience and willingness to participate again were the same and were included only in the questionnaire completed at the last study appointment (i.e., day 14 or day 21, study-dependent). In total, the four studies had 76 participants who completed their respective study and completed the relevant questionnaires (Table 1) in a private space. All 76 participants completed the questionnaires (100% response rate), and all questionnaires were analyzed. All procedures were carried out in accordance with relevant laws and institutional guidelines and have been approved by the appropriate institutional committee(s). Informed consents were obtained for the participants.

The principal investigator (Dimitris N. Tatakis) of the four studies, who performed the surgical interventions, was not involved in the distribution, explanation, or collection of questionnaires. The researcher who analyzed the deidentified questionnaires (Yi-Chu Wu) was not involved in the original trials and was blinded to recruitment processes, interventions, and trial clinical procedures and outcomes.

For study A [19], which included 20 participants (16 males and 4 females; aged 25.8 ± 2.1 years), two wounds, a 4 mm diameter and a 6 mm diameter, were created on each side of the palate with biopsy punches, that is, four wounds in total. Each wound was 1–1.5 mm deep. One side of the palate (both wounds) was randomly selected and received 10% phenytoin USP, while the contralateral wounds received carrier alone. Half of the participants received a biopsy of the smaller wound on day 1 and the other half on day 5 postoperatively; therefore, each participant received six wounding experiences, over two wounding appointments (study visits). Participant experience questionnaires were completed on day 21.

In study B [18], two wounds were created in each participant, one in each side of the palate. Prewounding with surgical incisions were made on one randomly chosen side of the palate 5 days prior to the actual excisional wound creation. Five days after prewounding, a standardized dimension free gingival graft was harvested from both sides of the palate (pristine side and prewounded side), resulting in a total of three wounding experiences for each participant, over two wounding appointments. The study included 10 participants (six males and four females; aged 26.4 ± 1.7 years) who completed the relevant questionnaires on day 21.

The 20 participants (14 males and 6 females; aged 32.5 ± 8.0 years) in study C [17] underwent standardized prewounding 3 or 5 days prior to connective tissue harvesting on one randomly selected side of the palate. Three or 5 days after the prewounding, a standardized connective tissue graft was harvested from each side of the palate (one pristine, one prewounded), also resulting in a total of three wounding experiences for each participant, over two study visits. Relevant questionnaires were completed on day 21.

For study D [20], which included 26 participants (12 males and 14 females; aged 28 ± 3.1), each participant received one standardized excisional wound (3 mm diameter biopsy punch, 1 mm depth) that was created in the buccal attached gingiva between the right maxillary premolars. No other intervention/wound treatment was applied. Relevant questionnaires were completed on day 14.

**Figure 1:**
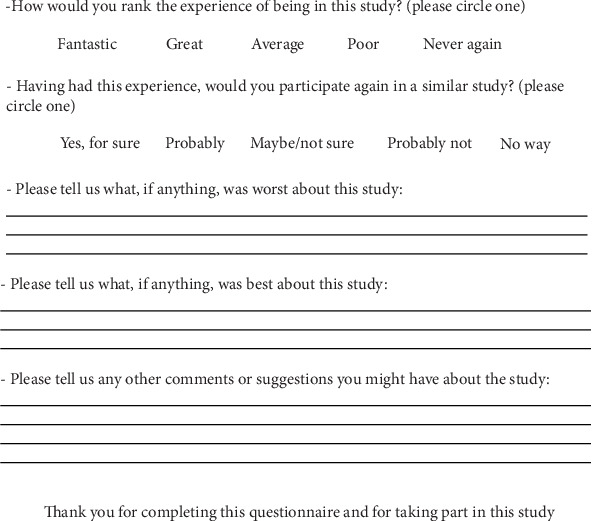
Questionnaire regarding study participation experience.

The relevant Likert-scale questions were *overall experience in the study* (*fantastic*, *great*, *average*, *poor*, and *never again*) and *willingness to participate in the study again* (*yes for sure*, *probably*, *maybe/not sure*, *probably not*, and *no way*). Additional open-ended questions addressed the *worst part about the study*, the *best part about the study*, and *other comments*. These specific questions are shown in Figure 1. Each participant contributed only one set of answers to the present study.

### 2.2. Data Management and Statistical Analysis

To evaluate the association between trial and participant's experience/willingness to participate again, frequency distribution analyses were performed using Fisher's exact test with Bonferroni correction for multiple comparisons. Logistic regression analysis was used to model the probability of rating experience as fantastic and of answering yes to repeat participation based on wound location and wound number. The association between experience and willingness to participate again was analyzed using a Fisher's exact test. Statistical significance was set at *p* < 0.05.

## 3. Results

**Figure 2:**
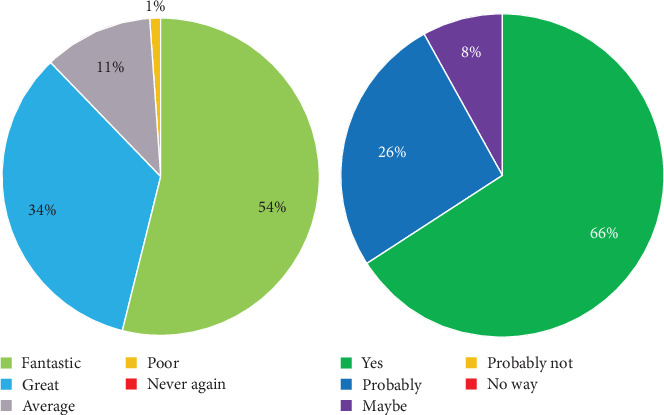
Overall responses to questionnaires. (A) Frequency distribution of responses regarding study participation experience. (B) Willingness to participate again. The reported data are percentage of respondents (total *n* = 76). Percentages were rounded to whole numbers.

All 76 participants answered the specific questions. On the question regarding experience of participating in a study, a great majority of volunteers (67; 88.2%) rated their experience as fantastic or good; only one person (1.3%) rated their experience as poor, and the rest (8; 10.5%) rated it as average. No participant rated their experience as never again (Figure 2A).

The responses regarding willingness to participate again were similarly distributed; the overwhelming majority responded positively (yes and probably, 50 [65.8%] and 20 [26.3%], respectively), and only 6 (7.9%) participants responded maybe. No participant responded probably not or no way (Figure 2B).

**Figure 3:**
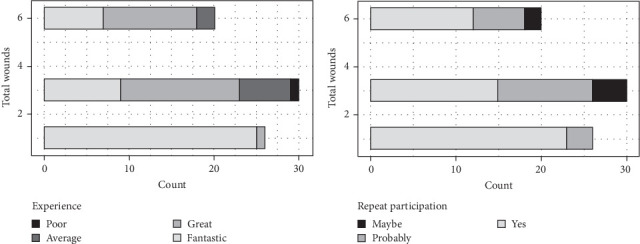
Responses to questionnaires in relation to total wound number. (A) Participation experience versus total wound numbers. (B) Willingness to participate again versus total wound numbers. The reported data are the number of participants (total *n* = 76). Differences between studies were significant for both responses (*p* < 0.001; Fisher's exact test with Bonferroni correction).

Of the 76 participants in the four trials, 50 had multiple (three or six) wounding experiences during the trial (all in the palate, trials A, B, and C), while 26 had only a single wound (on the buccal gingiva, trial D). Therefore, the number of wounds was chosen for further analysis. Trial participants who received only one wound reported either fantastic (25; 96%) or great (1; 4%) experience; no average or poor experience was reported. In contrast, 18% (*n* = 9) of trial participants who received more than one wound reported experience ranging from average to poor (Figure 3A). The difference between the four trials was significant (*p* < 0.001; Fisher's exact test with Bonferroni correction). When the four trials were sorted into two groups (one wound versus multiple), the difference between groups was also significant (*p* < 0.001; Fisher's exact test with Bonferroni correction); that is, the participants of the trials with multiple wounds (trials A, B, and C) had a significantly different experience than those in the single wound trial (trial D). When the multiple palatal wound trials were grouped into those with three or six wounding experiences, intergroup differences were not significant (*p* = 1).

When the answers regarding willingness to participate again were analyzed further, the results mirrored the participation experience responses. The 26 volunteers who received only one wound responded overwhelmingly positively, with 88.5% (*n* = 23) choosing yes for sure and 11.5% (n = 3) choosing probably. In contrast, among those who received 3 or 6 wounds, 54% (n = 27) chose yes for sure, 34% (n = 16) chose probably, and 12% (n = 6) chose maybe/not sure (Figure 3B). The difference between the four trials was significant (*p* < 0.001; Fisher's exact test with Bonferroni correction). However, the difference between the two groups of trials (one wound versus multiple wounds) was not significant (*p* = 0.097; Fisher's exact test with Bonferroni correction). The difference among the multiple palatal wound trials (3 or 6 wounding experiences) was also not significant (*p* = 1).

**Figure 4:**
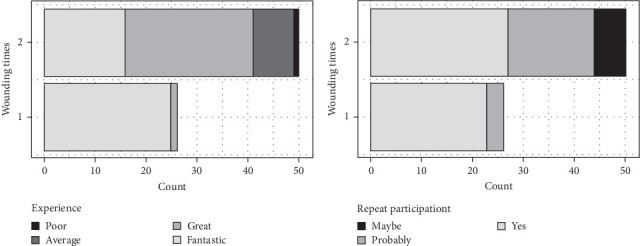
Responses to questionnaires in relation to the number of wounding times. Participation experience (A) and willingness to participate again (B) versus wounding times (appointments). The reported data are the number of participants (total *n* = 76). Differences between the number of wounding appointments were significant for both responses (*p* ≤ 0.033; Fisher's exact test).

Modeling probabilities were performed for participants' rating their experience as fantastic; the results showed that the number of wounding appointments (one or two appointments) was a determining factor (*p* = 0.0008) but not the total wound number (1, 3, or 6 wounds; *p* = 0.7106). The OR (95% CI) for rating the experience as fantastic by the number of wounding appointments (from one to two), while keeping the number of wounds constant, was 0.0109 (0.0002–0.3380; *p* = 0.0134), meaning that increasing the number of wounding appointments from one to two strongly decreases the odds of participants rating the experience as fantastic.  Figure 4A depicted patient experience by wounding times (one or two appointments). Among patients who experienced 2 wounding appointments, 16 patients reported fantastic, 25 reported great, 8 reported average, and 1 reported poor. For patient who experienced only 1 wounding appointment, 25 reported fantastic, and 1 reported great. There was a significant difference (*p* < 0.0001) among the two cohorts. When modeling probabilities were performed for participants' answering yes for sure regarding repeat participation, the results showed that the number of wounding appointments was a determining factor (*p* = 0.0118) but not the total number of wounds (*p* = 0.4879). Correspondingly, the cohort which experienced only one wounding appointment expressed more willingness to participate as a study individual again (*p* = 0.0327) (Figure 4B).

Furthermore, modeling probabilities were performed for participants' rating their experience as fantastic and for answering yes for sure regarding repeat participation based on the type of palatal wounding (punch versus graft; studies A–C); the results showed that type of wounding was not a determining factor for either response (*p* ≥ 0.4879).

**Figure 5:**
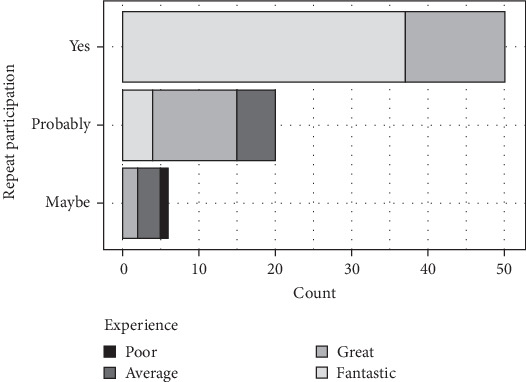
Willingness to participate again in relation to participation experience. The reported data are the number of participants (total *n* = 76). The participants' willingness to participate again in a study differed significantly based on their participation experience (*p*  < 0.001; Fisher's exact test with Bonferroni correction).

Overall, the participants' willingness to participate again in the study differed significantly based on their participation experience (*p* < 0.001; Fisher's exact test with Bonferroni correction), with 97% (*n* = 65) of those who had a positive experience (responses of fantastic or great) stating that they were likely to participate again (answering yes for sure [n = 50] or probably [*n* = 15]) (Figure 5). In contrast, none of the participants who did not have a positive experience (*n* = 9) answered yes for sure to repeat participation.

For the question about the worst part of the trial, the most frequent responses by the participants were the pain in the initial 1–3 days (*n* = 11), bleeding (*n* = 4) after the surgery, and the difficulty finding parking (*n* = 3) for the appointments. Regarding the best part about being a trial participant, most participants mentioned the easy access to the providers' schedule due to being part of the study (*n* = 10), the monetary compensation (*n* = 15), and the kindness and caring attitude of the research team members (*n* = 21).

## 4. Discussion

The purpose of this study was to evaluate the experience of participants in experimental periodontal wound healing studies and their willingness to participate in such a study again. The results showed that the participants reported overall satisfactory experiences, and most were inclined to participate again if the study became available again. The two responses were strongly correlated, with those who had a more positive participation experience being more likely to be willing to participate again. The results of this study, which is the first of its kind, indicate that participation in experimental periodontal wound healing studies, where participants do not receive a benefit from treatment, leaves participants with an overall positive experience.

There has been increasing awareness and interest in incorporating participants' experiences and perceptions as an important part of clinical trials. Nevertheless, limited relevant data have been reported on the topic, with most published studies being from the medical field [21–29]. To the best of the authors' knowledge, only two studies investigated participants' willingness to participate in dental clinical trials [15, 16]. More specifically, there have been no studies investigating the experience of participants in periodontal clinical trials or experimental studies. In the present study, the focus was on experimental wound healing studies; these studies shared several common characteristics: participants received standardized wounds, there was no direct treatment benefit to participants, and studies were conducted at the same institution. The reported overall positive experiences and willingness to participate again in such a study provide strong support for the implementation of future experimental studies. This is a significant finding, considering that in experimental wound healing studies, there is no direct benefit to participants, unlike what happens to patients participating in conventional periodontal intervention trials where they receive a treatment benefit. It would be of interest to have similar data reported from conventional periodontal intervention trials. The potential impact of having extensive informed consent forms—which included details of each study surgical procedures (including diagrams and clinical photos) and anticipated experiences from participation, including the lack of any potential direct benefit—on the reported overall positive experience of the participants merits future investigation.

The four clinical studies whose participants were included in the present analysis were chosen because they were similar in nature. They were studies where experimental wounds were created on maxillary masticatory mucosa sites (palate or buccal gingiva), and healing was followed over time after various interventions or without any intervention. The type of wounds and interventions were such that, for the most part, the participants experienced minimal pain; more specifically, of the 76 participants in the four studies, only two rated their postwounding pain as 1 out of 10 on the first postoperative day, with the remaining 74 reporting no pain (data not shown). It is likely that this limited pain experience of the participants affected their overall experience of being in the study and their willingness to participate again. The present study findings are not generalizable to conventional periodontal intervention trials, because of the different framework of these more routine trials when compared to the experimental studies investigated here. This lack of generalizability of the findings is a limitation of the present study. Another study limitation is the fact that, due to the nature of the available studies, it was not possible to differentiate between participants with one wound from those with a buccal wound; this prevented a separate analysis between participants receiving buccal wounds and those receiving palatal wounds. Nevertheless, the present study strengths include the large number of participants and the consistent approach (identical questions, always given at last study visit) used to assess participants' experience in each study.

Differences among the studies included the number of wounds performed and the number of wounding visits (appointments) for each study. Therefore, further analysis of the participants' opinion of their experience was performed based on number of wounds and number of wounding appointments. The present study findings indicate that the number of wounds received has a strong effect on participants' overall experience as well as their willingness to participate again. The determining factor for participants expressing very high satisfaction with their participation experience was the number of wounding appointments. These finding suggest that an experimental wounding study with less surgical intervention visits is more likely to result in more satisfied participants. This novel finding may be helpful to investigators considering the design of future experimental wounding studies.

There is scarce information regarding participants' willingness to be part of a clinical study. Most of these limited studies were conducted in the general medical field. It has been reported that the patients' major concern of taking part in a clinical trial is the safety of the proposed treatment [21]. However, when there was more information presented to the patients, they were more likely to participate in the trial [21, 22]. In order to disseminate such information and to increase the awareness of ongoing or planned clinical trials, it had been shown that mass media, television, Internet, and hospital advertisements are often used [21]. Nevertheless, even with an affirmative awareness of 75% or more [21, 23], only about 25% of respondents are willing to participate in a clinical trial.

The reported low rate of willingness to be part of a clinical study inevitably raises questions regarding the factors that drive respondents' decision-making. Many studies point out that the trust of the clinician and care team is critical for participants' decision [25–27]. In the present study, more than one third of the participants answered what was the *best part about the study* and offered *other comments* recognizing the members of the research team. The second most frequent mention as *best part about the study* was the monetary compensation. This finding is in agreement with the established incentive of clinical trials [16, 28–30] and echoes the known barrier to clinical trial participation, that is, the out-of-pocket cost of the offered treatment [24]. Other common reasons for patients to participate in clinical studies include the favorable feeling of being in a trial [21], benefit of a better treatment [25], or feeling of receiving better care [26]. In the present analysis, some participants highlighted the fact of easier appointment process as well as dedicated personnel for scheduling. Lastly, contribution to science and pure altruism was also mentioned by the participants, which is in common with reported medical studies [25].

The significance of the present study findings is that, even in the absence of any therapeutic benefit, the majority of participants in periodontal experimental wound healing studies have a positive experience and would be willing to participate again in such a study. This affirmative finding should be encouraging to investigators planning on pursuing future studies of this nature and should also facilitate the approval of such research protocols by the institutional review boards. Just as it is important to incorporate patient views in the course of efforts to improve healthcare [31], incorporation of participant views in clinical research studies should help improve study processes and designs. Wensing and Elwyn [31] highlight the fact that patients can and should give their preferences for care and offer evaluations of what transpired. The very limited information available on participant experiences in dental trials, in general, and in periodontal trials, in particular, strongly suggests the need for future research on the specific topic. The questions asked of the participants from the periodontal experimental studies analyzed herein addressed participants' experiences and offered them the opportunity to provide open-ended comments on the positive and negative aspects of their experience. The present study findings are the first results to attempt to narrow the knowledge gap on periodontal study participants' experiences and should help improve future study designs and, consequently, improve volunteer recruitment. The hope is that such efforts will ultimately lead to improved periodontal care for our patients.

Within the limitations of the present study, volunteers participating in periodontal experimental wound healing studies had an overall highly positive experience and expressed willingness to participate again, with the number of wounds received and number of wounding visits affecting participants' responses. There is a need for additional research on the experience of periodontal clinical intervention trial participants.

## Figures and Tables

**Table 1:** Characteristics of the included experimental wound healing studies.

Study	Wound site	Wounding visits	Total wounds created	Participants
A	Palate	2	6	20
B	Palate	2	3	10
C	Palate	2	3	20
D	Buccal gingiva	1	1	26

## Data Availability

The data that support the findings of the study are available on request from the corresponding author.
